# A biomechanical model of anther opening reveals the roles of dehydration and secondary thickening

**DOI:** 10.1111/j.1469-8137.2012.04329.x

**Published:** 2012-09-21

**Authors:** M R Nelson, L R Band, R J Dyson, T Lessinnes, D M Wells, C Yang, N M Everitt, O E Jensen, Z A Wilson

**Affiliations:** 1 School of Mathematical Sciences University of Nottingham NottinghamNG7 2RD UK; 2 Centre for Plant Integrative Biology School of Biosciences University of Nottingham Sutton Bonington NottinghamLE12 5RD UK; 3 School of Mathematics University of Birmingham BirminghamB15 2TT UK; 4 Mathematical Institute University of Oxford24-29 St Giles' Oxford OX1 3LB UK; 5 Faculty of Engineering University of Nottingham NottinghamNG7 2RD UK; 6 School of Mathematics University of Manchester Oxford Road ManchesterM13 9PL UK

**Keywords:** actuation, anther, biomechanical modelling, dehiscence, dehydration, secondary thickening

## Abstract

- Understanding the processes that underlie pollen release is a prime target for controlling fertility to enable selective breeding and the efficient production of hybrid crops. Pollen release requires anther opening, which involves changes in the biomechanical properties of the anther wall. In this research, we develop and use a mathematical model to understand how these biomechanical processes lead to anther opening.
- Our mathematical model describing the biomechanics of anther opening incorporates the bilayer structure of the mature anther wall, which comprises the outer epidermal cell layer, whose turgor pressure is related to its hydration, and the endothecial layer, whose walls contain helical secondary thickening, which resists stretching and bending. The model describes how epidermal dehydration, in association with the thickened endothecial layer, creates forces within the anther wall causing it to bend outwards, resulting in anther opening and pollen release.
- The model demonstrates that epidermal dehydration can drive anther opening, and suggests why endothecial secondary thickening is essential for this process (explaining the phenotypes presented in the *myb26* and *nst1nst2* mutants).
- The research hypothesizes and demonstrates a biomechanical mechanism for anther opening, which appears to be conserved in many other biological situations where tissue movement occurs.

## Introduction

**Figure 1:**
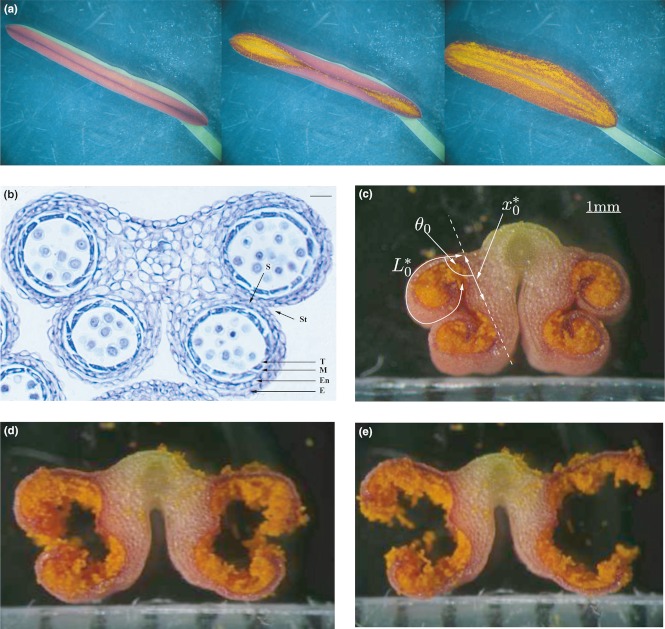
(a) Sequence of developmental stages and configurations observed during anther opening (lily). (b) Cross-section of an anther (Arabidopsis) showing the four distinct locules containing the developing pollen grains within; the four cell layers of the maternal anther (E, epidermis; En, endothecium; M, middle cell layer; T, tapetum) are present at this stage, and anther opening occurs at the point of the stomium (St) and septum (S). Bar, 20μm. (c) Cross-section of a closed anther (lily) once the middle cell layer and tapetum have degraded, and the septum and stomium have split. The geometric parameters required by this model are illustrated: the total arc-length in the closed configuration, 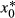) and angle (θ_0_) of the fixed support. (d,e) Increasingly open configurations of the lily anther shown in (c).

Pollen is formed within specialized structures (stamens) within the flower, which comprise an anther (Fig. 1a), containing the pollen, and a filament, which provides the vascular connections to the flower and allows the anther to be presented away from the floral centre. Pollen development occurs centrally within the anther locules, which are surrounded by four maternal cell layers: the outer epidermis, the endothecium, the middle cell layer and the tapetum (Fig. 1b). After microspore meiosis, the tapetum and middle cell layer degenerate, whereas the endothecium undergoes selective deposition of secondary thickening (Scott *et al*., 2004; Ma, 2005). Anther dehiscence and pollen release involve a number of distinct phases, including deposition of secondary thickening in the endothecium, enzymatic digestion of cell walls at the septum between the two locules, pollen swelling, differential endothecial and epidermal cell expansion and, finally, dehydration. These processes lead to stomium splitting and retraction of the anther walls and subsequent pollen release (Fig. 1a; Supporting Information, Video S1; Wilson *et al*., 2011). Anther dehiscence is highly regulated, enabling the timing of pollen release to be tightly controlled to maximize the chances of fertilization.

Breakage of the septum and stomium is essential for anther dehiscence. Initially degeneration of the septum occurs, generating a bilocular anther, which is followed by stomium cell breakage and then retraction of the anther wall and pollen release (Fig. 1a). These breakages are associated with a number of biomechanical changes within these cells, which are thought to involve cell wall-degrading enzymes (which break down the pectin in the cell walls) and programmed cell death (Sanders *et al*., 2000; Wilson *et al*., 2011). There have been a number of reports of dehiscence mutants resulting from changes to stomium degeneration, which indirectly inhibit breakage of the septum and stomium, although the pollen appears normal (Dawson *et al*., 1999; Sanders *et al*., 1999; Mitsuda *et al*., 2005; Yang *et al*., 2007).

Dehydration has been implicated as essential to the process of anther opening, with the general requirement being removal of the central locular fluid before pollen release and dehydration of the anther wall (Pacini *et al*., 2006). Anthers appear to go through a process of targeted dehydration that may involve both evaporation through stomata on the epidermal surface and also selective removal of water from the anther (Keijer & Cresti, 1987). Although relative humidity rates have an effect on anther opening (Keijer & Cresti, 1987), it seems evident that the dehydration process is also an active process. It has been shown in tomato that there are regions of differential hydration within the anther, with conversion of starch to sucrose occurring selectively in anther connective tissues, which would result in an increased osmotic potential with the effect of removal of water from the anther (Bonner & Dickinson, 1989). There have also been other reports of water status changes within the anther, including high concentrations of the H^+^-ion sucrose transporter (AtSUC1) accumulating in *Arabidopsis* anther connective tissues (Stadler *et al*., 1999) and increased levels of *Petunia NECTARY1 (NEC1)* and *NEC2* (which function in starch to sugar regulation) in the filament and stomium (Ge *et al*., 2000, 2001). Aquaporins have also been reported to affect anther opening. The aquaporins are a large gene family of membrane proteins associated with cell-to-cell movement of water in different tissues (Tyerman *et al*., 2002). In tobacco, two aquaporins, PIP1 and PIP2, have previously been shown to accumulate preferentially in the anther and stylar tissues (Bots *et al*., 2005a,b). Aquaporin accumulation has also been linked to pollen dehydration and subsequent rehydration upon contact with the stigmatic surface (Ruiter *et al*., 1997; O'Brien *et al*., 2002; Soto *et al*., 2008; Pacini *et al*., 2011). These reports and the observation that open lily anthers can be induced to close by wetting of the anther wall surface (Z. A. Wilson and C. Yang, unpublished) indicate the critical importance of dehydration during anther dehiscence.

Endothecial secondary thickening is also critical for anther dehiscence; *Arabidopsis* mutants that lack endothecial secondary thickening fail to dehisce and release pollen, making them effectively male sterile, although the pollen produced is fully viable (Dawson *et al*., 1999; Mitsuda *et al*., 2005; Yang *et al*., 2007). In the *Arabidopsis myb26* mutant, anther development initially appears to be normal, and after meiosis the tapetum and middle cell layer start to degrade; however, the endothecial layer fails to expand and the secondary thickening seen in the wildtype anther endothecium does not form (Dawson *et al*., 1999). Pollen development and subsequent septum degradation appear to occur normally but, as the anther dehydrates, the endothecial cells collapse, resulting in failure of retraction of the anther walls and a lack of pollen release (Dawson *et al*., 1999). In the wildtype, secondary thickening is highly specific, occurring in the endothecium, but not in the epidermal cell layer. If thickening is ectopically induced in the epidermis, as a consequence of misexpression of *MYB26*, the anthers also fail to open and the lines are also effectively male sterile (Yang *et al*., 2007).

While there is strong evidence that the properties of the endothecium allow anther opening, we are not aware of any previous biomechanical study of this process. As is typical in plant cells, the cells of the anther are surrounded by a fibrous cell wall, which sustains a high internal cell turgor pressure (Dumais & Forterre, 2012). This cellular structure enables plant tissue to withstand mechanical forces, and yet move and grow in response to changes in turgor and cell wall properties. Cell walls consist of cellulose microfibrils, embedded within a pectin–hemicellulose matrix (Cosgrove, 2005). As is seen in the anther's endothecial cells, the cellulose microfibrils and the lignified secondary thickening are typically orientated in a preferred direction, causing cell walls to be mechanically anisotropic (i.e. much less extensible in a direction parallel to the cellulose fibres than perpendicular to them) (Baskin, 2005; Dyson & Jensen, 2010).

Changes in turgor arise as a result of both passive dehydration and active regulation of the cell's osmotic potential. Such changes can cause small differences in cell volume, which, owing to the geometry and structure of the tissue, can lead to large reversible movements at the organ scale (Dumais & Forterre, 2012). A number of recent studies have shown that if the cellulose orientation (or amount) varies between different cell layers, changes in turgor can lead to unequal shrinkage of the cell layers, which can cause the tissue to bend. For example, the scales of pine cones have a bilayer structure: the outer layer has cellulose fibres orientated perpendicular to the scale that lengthen/shrink in response to changes in humidity, whereas the inner layer does not respond as strongly. As the humidity of the environment changes, this structure causes the scale to bend, resulting in the pine cone opening and closing (Dawson *et al*., 1997; Reyssat & Mahadevan, 2009). Similar mechanisms have been shown to be key to the opening of chiral seed pods (whose valves consist of two fibrous layers each orientated at *c*. 45° to the pod's longitudinal axis; Armon *et al*., 2011), and to cause the circadian opening and closing of the awns of wild wheat seeds (in which the inner layer has organized cellulose and the outer layer has randomly orientated cellulose; Elbaum *et al*., 2007).

Here, we present a mathematical model of anther dehiscence that describes the biomechanics of anther opening. The model describes how epidermal dehydration can drive anther opening and demonstrates why secondary thickening of the endothecium is essential for this process. Although the mechanisms described are relevant to many species, we consider here oriental lily (*Lilium*) and *Arabidopsis thaliana* anthers, since these are commonly studied experimentally.

## Description

To gain an understanding of the biomechanics of anther opening, we developed a two-dimensional mathematical model of the cross-section of an anther, neglecting any variations along its axis. We consider an anther in which the tapetum and middle cell layers have degraded, the endothecium has undergone secondary thickening, and the stomium and septum have broken (Fig. 1c). We therefore model the anther wall as two cell layers: the endothecium and the epidermis (Fig. S1). Dehydration of the epidermal cells will reduce their turgor pressure, reducing the natural (unstressed) length of the epidermis; the stiffer endothecium does not contract appreciably. We assume that the epidermis is tightly adhered to the endothecium, so that the two layers remain approximately the same length. Differential contraction of the two layers results in the bilayer having a preferred curvature that evolves with dehydration of the epidermis, causing bending (Fig. d,e). To enable this to happen, secondary thickening inhibits contraction of the endothecium and provides resistance to bending. The model predicts how the interplay between continuing dehydration of the epidermis and resistance to bending as a result of endothecial secondary thickening controls anther opening and pollen release. The focus here is on the role of epidermal dehydration, although it is not known whether the endothecium also dehydrates; we neglect endothecial dehydration in what follows.

We restrict attention to one representative pair of locules, assuming symmetry about the (former) site of the septum. We suppose that tissue at the base of the locule pair (demarcated by the straight line in Fig. 1c) remains unaltered during anther opening, an assumption that is consistent with configurations observed experimentally, and that the locule base has a fixed width, 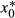 , and makes a fixed angle, *θ*_0_, with the free anther wall. We assume that the pressure difference acting across the anther wall is negligible; configurations are therefore determined by the properties of the anther wall alone.

The mathematical equations governing anther opening are described in detail in Notes S1, and details of a similar biomechanical model for a mammalian epithelial layer can be found in Nelson *et al*. (2011). The shape of the anther wall is governed by force and moment balance equations for the endothecium. Between the endothecium and epidermis act tangential and normal forces (Fig. S2), which depend upon the tension within the epidermis and the curvature of the anther boundary, transmitting the effects of epidermal dehydration to the endothecium. The force and moment balance equations for the endothecium describe: how the tangential stress within the endothecium is balanced by the tangential stress from the epidermis; how the normal stress within the endothecium is balanced by the normal reaction from the epidermis; and how the tangential stress from the dehydrating epidermis generates bending of the endothecium.

We make simple constitutive assumptions for the endothecium and epidermis. Supposing that strains are sufficiently small, we model the cell walls as elastic materials so that within each layer the tangential stress is assumed to be proportional to the difference between the current strain and the natural resting strain, with the constant of proportionality being an extensional stiffness parameter. This constitutive relationship, and how it relates to the cell-scale properties, can be derived from a force balance on an individual cell. For an epidermal cell, the extensional stiffness parameter is proportional to the cell wall's extensional stiffness, with the resting strain of the epidermis depending on the turgor pressure. As the epidermal turgor pressure reduces, because of dehydration, the resting strain also reduces, causing either an increase in tangential stress or a reduction in length. To determine the extensional properties of the endothecium, we take into account the effect of the secondary thickening. This comprises stiff lignocellulose fibres that form a helix around each endothecial cell (Garcia, 2002; Yang *et al*., 2007). The secondary thickening acts in concert with the cell wall and turgor in determining the properties of the endothecial cells. Using a formula for the extensional stiffness of a helix (Costello, 1977), we find that the contributions to the extensional stiffness and resting strain of the endothecium from secondary thickening depend on the radius, Young's modulus and Poisson ratio of the fibres, the pitch angle of the helix and the endothecial cell thickness. In particular, this demonstrates that the secondary thickening provides a high resistance to stretching of the endothecium.

We also require a constitutive assumption for the bending moments. Because of the helical secondary thickening within the endothecial cell walls, the endothecium resists bending, with the bending moments being assumed proportional to the endothecial curvature. As for the extensional stiffness, secondary thickening causes the constant of proportionality (the bending stiffness) to depend upon the radius, Young's modulus and Poisson ratio of the fibres and the pitch angle of the helical spring (Costello, 1977). By contrast, as there is no secondary thickening of the epidermis, we suppose that its bending resistance is negligible.

Having defined the mechanical properties of the anther wall, it remains to prescribe boundary conditions at its ends. As the anther opens, it moves through three different configurations (Fig. S3) and we specify appropriate boundary conditions to each case. In case I, we consider a closed anther whose walls are tightly curled, such as that of Fig. 1(c). Tangential forces are zero at the symmetry line between the two locules, and since no external forces act upon the anther wall in the curled-under region, the anther wall attains its preferred curvature here; the associated configuration is simply an arc of a circle if the preferred curvature is uniform. As the anther dehydrates, the anther walls uncurl and the point of contact between the two locule walls moves towards the tip of each wall (Fig. S3a,b). Configurations for which the two locules touch at their tips are described by case II, for which the anther remains closed. Boundary conditions are similar to case I; however, all forces at the contact point now act perpendicular to the line of symmetry. In both closed configurations (cases I and II), we monitor the contact force between the locules, which decreases as continued dehydration results in further reduction in the preferred curvature of the anther wall. Once the contact force reduces to zero, the anther opens (case III). Under case III, there is no external force upon the anther wall and the preferred curvature is attained uniformly; configurations are simply arcs of circles if the preferred curvature is uniform (Fig. S3c), although individual locules may, in practice, show some deviation from this idealized configuration (Fig.d,e).

The model predictions depend on the geometry of the locule, material properties of the two layers (extensional stiffnesses and bending stiffness of the endothecium) and the natural lengths of the two layers. These parameters and appropriate estimates for lily and *Arabidopsis* anthers are presented in Table 1. Geometrical parameters were measured from images of fresh and fixed lily and *Arabidopsis* anther cross-sections; the estimate of endothecial bending stiffness comes from a simple ‘weight-lifting’ experiment, described in Notes S2. As described in the Supporting Information, the governing equations can be nondimensionalized such that they depend only upon eight dimensionless groupings of dimensional parameters, summarized in Table 2. The data in Table 1, together with cell-scale arguments given in Notes S1 (Section 1.4: Reduced model in the inextensible limit) and Table S1, enabled us to estimate the magnitudes of key parameter groupings, which describe key ratios of mechanical properties. We highlight, in particular, three important quantities: 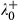, which represents the resting strain of the epidermis, which falls during dehydration; *β*, which measures the relative extensibilities of the epidermal and endothecial layers; and *Φ*, which measures the capacity of epidermal shrinkage to generate endothecial bending.

**Table 1:** Physical parameters of the biomechanical model

Parameter	Symbol	Estimated values
Lily	*Arabidopsis*
Natural length of the anther wall segment	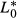	2 mm	0.19 mm
Width of the locule	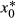	0.67 mm	0.06 mm
Angle at the support	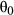	2π/3 – π
Half of the endothecium thickness	h*	0.05 mm	4 μm
Bending stiffness of the endothecium	D*	2 × 10^−6^ Pa m^3^
Preferred curvature of the endothecium	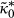	Assumed zero
Extensional stiffness of the epidermis	*k*^+^*	Unknown
Extensional stiffness of the endothecium	*k*^−^*	Unknown

Estimates of geometric parameters are based on measurements taken from 17 mature lily anthers and eight mature *Arabidopsis* anthers. Endothecial bending stiffness is estimated via an experiment described in Supporting Information Notes S2.

**Table 2:** Dimensionless parameters

Parameter	Symbol	Formula	Estimated value
Resistance to endothecial extension relative to endothecial bending	*α*	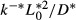	≫ 1
Resistance to epidermal extension relative to endothecial extension	*β*	*k*^+^*/*k*^−^*	≪ 1
Resistance to epidermal extension relative to endothecial bending	*Φ*	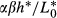	O(1)
Preferred curvature of the endothecium, scaled on locule wall length	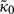	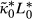	0
Resting strain of the epidermis	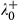		Varies as a function of hydration
Resting strain of the endothecium	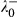		1
Width of the locule relative to the length of its wall	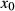	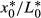	1/3
Angle at the support	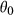		2π/3 – π

For estimates of magnitude, refer to Supporting Information Notes S1 (Section 1.4: Reduced model in the inextensible limit).

## Results

**Figure 2:**
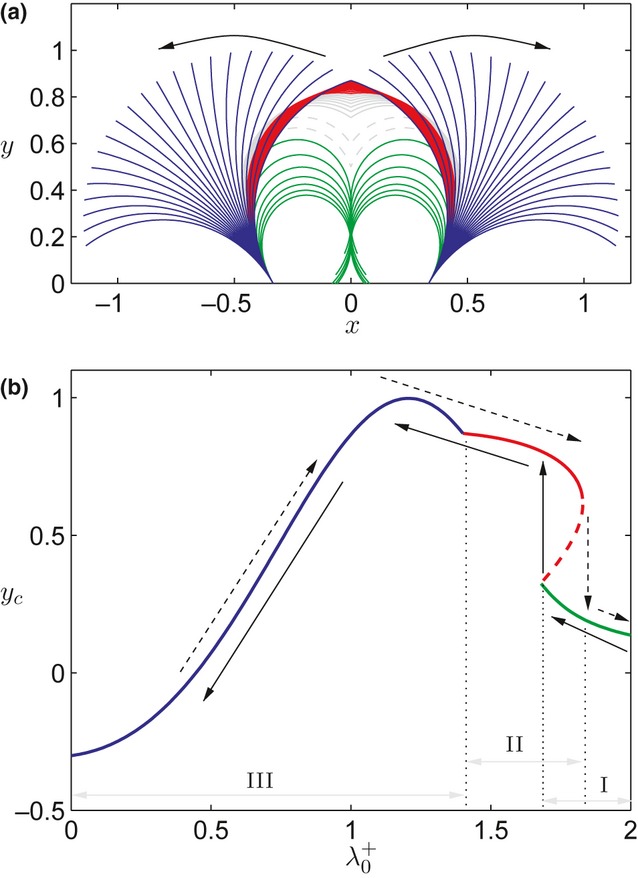
(a) Configurations predicted with parameters appropriate for lily and *Arabidopsis* anthers as epidermal dehydration reduces turgor and hence 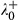. Arrows indicate 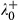 decreasing from 1.9 to 0.56. (b) The same configurations characterized instead by the *y*-coordinate at the tip of the anther wall as a function of 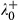. In the absence of the septum, the locules initially lie in a closed, tightly curled ‘case I’ configuration, which gradually uncurls as the epidermis dehydrates (green curves). For 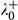 ≍ 1.66, a snap-through transition to ‘case II’ occurs (red solid curves), at which point the anther wall is uncurled but the anther still remains closed. Case II solutions evolve until 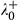 ≍ 1.4, at which point a transition to ‘case III’ occurs and the anther opens (blue curves). In case III, anther walls are arcs of circles, which uncurl as 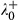 decreases. For these parameters, hysteresis occurs at the transition from case I to case II; the sequence of configurations attained by a dehydrating anther (illustrated by solid arrows in (b)) is slightly different from those seen during rehydration of an already open anther (dashed arrows in (b)). In panel (a), grey shapes represent mathematically viable case II solutions (solid, stable; dashed, unstable) that would not be attained by a dehydrating anther. Parameters used: *α* = 1000, *β* = 0.2, 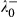 = 1, *Φ* = 5, 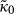 = 0 *x*_0_ = 1/3, *θ*_0_ = 2π/3.

Simulating the model using MATLAB, we can predict how changing the turgor pressure within the epidermis drives the dynamics of anther opening. Fig. 2 illustrates the configurations predicted by the model with different values of the epidermal turgor pressure (using the parameter values appropriate for the lily anther given in Table 2). Associated distributions of stress and strain are plotted in Fig. S4. As the epidermal cells dehydrate, their turgor reduces, and we predict that the anther wall progressively moves through this sequence of configurations. Initially, after septum breakage, the closed anther exhibits a case I configuration, with the free ends of the anther wall tightly curled at the point of contact between the locules (green curves, Fig. 2a). The model predicts that, as the epidermis dehydrates, the anther wall uncurls (remaining closed) until the tips of the locule walls are in contact. At this point a snap-through transition to case II occurs, the next configuration being that of a solid red curve in Fig. 2(a). To further clarify these dynamics, Fig. 2(b) shows the *y*-coordinate of the anther wall at the symmetry line as a function of the hydration parameter, 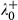 (which decreases proportionately to the epidermal turgor pressure); the sequence of configurations attained by a dehydrating anther are shown by solid arrows. As the figure illustrates, for values of 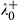 between *c*. 1.66 and 1.8 (equivalent to a range of epidermal hydration), there exist three possible solutions: a stable case I solution (solid green line) and two case II configurations, one unstable (dashed red line) and one stable (solid red line). During dehydration, at the transition from case I to case II, the anther adopts the only available stable configuration (which lies on the solid red curve in the figure). Continued epidermal dehydration reduces the contact force at the symmetry line to zero, at which point the anther opens (case III, blue curves). Further epidermal dehydration results in the anther opening progressively wider, recovering (at least approximately) the shapes illustrated in Fig. 1(d,e). Rehydration of a fully open anther is equivalent to traversing Fig. 2(b) from left to right, as marked by the dashed arrows. Close to the transition from case II to case I, the anther remains in a case II configuration for higher values of 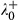 (when compared with the dehydrating configurations), jumping from the red curve back to the green curve for 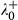 = 1.8.

Fig. 1(a) illustrates that, on opening, a lily anther gradually unpeels from its tips, the central portion of the anther opening last. Measurements of a lily anther in closed and open configurations suggest that while the width of the closed anther is approximately uniform along its length, the width of the locule wall (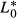) falls to approximately half of its maximum value near the tips. The model demonstrates how the parameter 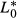 affects the degree of epidermal dehydration required for the anther to open, as discussed in the Supporting Information (Notes S3). As Fig. S5 shows, the model predicts that a decrease in 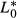 leads to transitions to open configurations for larger values of 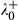, that is, lesser epidermal dehydration. This suggests that dehiscence initially occurring near the anther tips arises as a consequence of the shape of the anther wall.

**Figure 3:**
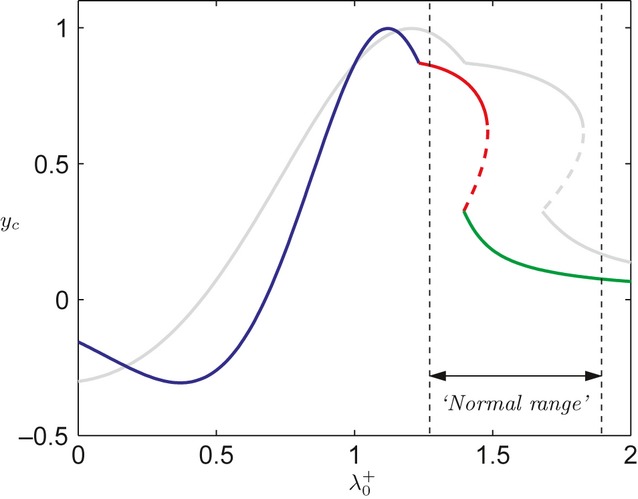
configurations attained for mutants with a stiffened epidermis or reduced endothecial secondary thickening (coloured curve), compared with those of a wildtype anther given in Fig. 2(shown in grey). The dominant parameter controlling the transition from closed to open states is *Φ*; for larger *Φ*, the solution curve is squashed horizontally towards 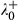 = 1, requiring stronger dehydration for the anther to open. Also illustrated is a speculated normal range of hydration. Parameter changes that push the transition from case II to case III outside the biologically feasible range correspond to anthers that fail to open. Parameters used for mutants: *β* = 0.4, *Φ* = 10, and all other parameters as in Fig. 2.

We now consider the extent to which biomechanical changes impact the anther's ability to open. Anther dehiscence has been observed to be hindered by both reduced endothecial secondary thickening, such as that observed in the *myb26* and *nst1nst2* mutants (Dawson *et al*., 1999; Mitsuda *et al*., 2005), and stiffening of the epidermis through, for example, ectopic deposition of secondary thickening (Yang *et al*., 2007). In the former case, the endothecial extensional and bending stiffnesses are reduced by the same factor, whereas in the latter case the epidermal extensional stiffness is increased. Thus, considering the dimensionless parameter groupings in Table 2, both cases cause an increase in the parameter *β* and an associated proportional increase in the parameter *Φ*. Since *α* ≫ 1 and *β* ≪ 1, we find (see Notes S1: Reduced model in the inextensible limit) that the behaviour of the anther is dominated by the parameter *Φ*, such that an increase in *Φ* simply shrinks solution curves in Fig. 2(b) with respect to the horizontal axis about the point 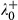 = 1. Fig. 3 illustrates the solution branches obtained for *β* = 0.4 and *Φ* = 10 (double the values used in Fig. 2), with all other parameters as in Fig. 2. (The predicted wildtype behaviour is marked in grey for comparison.) The increase in *Φ* results in solution curves being pushed towards 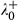 = 1 in Fig. 3, with anther opening (the transition from red to blue curves) now requiring greater epidermal dehydration (smaller 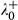). Also illustrated in Fig. 3 is a speculated ‘normal range’ of 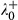 (equivalent to epidermal turgor) in a healthy anther. Changes in *Φ* that result in the transition from case II to case III (red to blue) falling outside this healthy range correspond to anthers which would fail to open under normal conditions.

As a means of model validation, experiments were conducted in which mature lily anthers were compressed by an applied load in order to estimate the forces associated with dehiscence. Full details are given in Notes S2. The experiments conclude that an applied load of 0.01 N is sufficient to prevent the locule from opening. Experimental and theoretical estimates (given in the Notes S2) both give the bending stiffness of the anther wall as *c*. 2 × 10^−6^ Pa m^3^, providing a consistency check of the model.

## Discussion

The biomechanical model presented here supports and explains the hypothesis that dehydration of the cells of the anther walls provides the force required for anther dehiscence. The model demonstrates that endothecial secondary thickening plays a crucial role in this mechanism, by providing differential resistances to bending and stretching, and hence allowing the forces of dehydration-driven epidermal contraction to change the anther's shape.

In this model, simple cell-scale arguments were used to relate evolving epidermal turgor pressures to changes in the resting strain of the epidermis. The model demonstrates that the configurations adopted by the anther are controlled by: changes in epidermal hydration (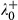), which drive changes to the preferred curvature of the bilayer; the ratio of endothecial resistances to bending and stretching, which is affected by endothecial secondary thickening (*α*, see Table 2); the relative resistances to extension of the epidermis and endothecium (*β*); and the resistance of epidermal extension relative to endothecial bending (*Φ*). In the limit of large *α* and small *β*, behaviour is dominated by the material parameter *Φ*, variations in which control the degree of dehydration required to allow the anther to open. The model demonstrates that anther dehiscence is hindered by either a reduction of endothecial secondary thickening or an increase in the stiffness of the epidermis (both of which proportionally increase *β* and *Φ*; Fig. 3). Alteration of these are both effectively observed in *myb26* mutants and overexpression lines; knockouts of the *MYB26* gene have been shown to reduce endothecial secondary thickening (Dawson *et al*., 1999; Yang *et al*., 2007), while overexpression of the gene stimulates secondary thickening in the epidermis (Yang *et al*., 2007). In each of these lines, the anther fails to open, or shows partial opening, and pollen cannot be released.

The model illustrates that epidermal dehydration can drive transitions from closed configurations (case I, Fig. 1c) to open (case III) configurations such as those of Fig. 1(d,e). For a uniform preferred curvature, the model predicts open configurations to be simply arcs of circles (Fig. 2); however, Fig. 1(d,e) illustrates that the biological configurations exhibit some localized deviations from this idealized situation, and shows that variations occur between locules. A more detailed understanding of the processes underlying dehydration would be required in order to capture accurately these variations in preferred curvature. Fig. 1(a) illustrates that the anther is widest at the centre, narrowing at the tips; the model shows that these variations (at least partially) explain why the anther initially opens near the tips (Fig. S5).

The model demonstrates how the passive mechanical properties of plant tissue can generate movements, suggesting that the bending of the anther wall is the result of a similar mechanism to those proposed for pine cone scales (Dawson *et al*., 1997; Reyssat & Mahadevan, 2009) and wheat awns (Elbaum *et al*., 2007) (see Introduction). In contrast to these examples, during anther opening, the geometry of the anther wall is constrained and it cannot always attain its preferred curvature. The model presented here predicts that gradual dehydration causes a build-up of stress within the anther wall, leading to a snap-through transition to a new configuration, at least for the geometric and material parameter values associated with lily and *Arabidopsis* anthers. Such snap-through behaviour is dependent upon specific values of the geometric parameters 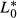, 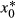 and *θ*_0_ (Fig.c), which may vary across other species. Thus, the model demonstrates how gradual dehydration can lead to movement, although the model does not predict the speed at which this happens. The potential importance of such actuation systems to inspire biomimetic devices has recently been reviewed (Burgert & Frazl, 2009) and may lead to the development of materials that move in response to environmental changes with minimal energy requirement.

In presenting our mechanism of anther opening, we focused on the role of passive dehydration; however, the mechanism could also apply to, and be controlled by, active processes. Osmotic changes in the epidermal cells could drive water flows, and potentially could control turgor variations along the epidermal layer, providing spatial regulation of the anther wall's preferred curvature. Furthermore, the rate at which water flows across cell membranes could be controlled by the content of aquaporins, which increase the membrane permeability. As discussed in the, several studies of anther tissues have reported specific patterns of sucrose (Bonner & Dickinson, 1989) and aquaporin accumulation (Bots *et al*., 2005a,b). These observations imply that a highly selective process of dehydration is occurring at defined points during plant reproduction (Bonner & Dickinson, 1990).

Our mathematical model for the opening of the anther provides support for the importance of the bilayer system for anther opening and illustrates the interaction between selective dehydration of the epidermis and the secondary thickening of the endothecial cell wall. Data obtained from observation of transgenic lines in which the amount of secondary thickening is altered in the endothecium and in the epidermis support the proposed mechanism; further work is planned to determine how anther dehiscence is affected by altering the dehydration status by transgenic modification.

## Supporting Information

**Fig. S1** Schematic diagram of the biomechanical model.

**Fig. S2** Distribution of forces and moments upon an arbitrary bilayer segment.

**Fig. S3** The three classifications of geometric configuration modelled.

**Fig. S4** Stresses and strains associated with the configurations of Fig. 2.

**Fig. S5** Critical value of epidermal hydration required for a transition to an open configuration, as a function of L*_0_.

**Table S1** Cell-scale parameters

**Notes S1** The model.

**Notes S2** Estimating forces of anther dehiscence.

**Notes S3** The effects of variations in L*_0_.

**Video S1** Video of opening of lily anther (showing 220 frames taken once every 10 min, played back at 10 frames s^−1^).
