# Supplementary material for: A biomechanical model of anther opening reveals the roles of dehydration and secondary thickening
Source: New Phytol. 2012 Sep 21;196(4):1030–7. doi: 10.1111/j.1469-8137.2012.04329.x (PMC3569878; doi:10.1111/j.1469-8137.2012.04329.x)
Supplement: Supplementary file 1 [file nph0196-1030-SD1.pdf]

A biomechanical model of anther opening reveals the roles of dehydration and secondary thickening

M. R. Nelson, L. R. Band, R. J. Dyson, T. Lessinnes, D. M. Wells,
C. Yang, N. M. Everitt, O.E. Jensen, Z. A. Wilson

August 2012

Supporting Information

Notes S1: The model

We formulate a two-dimensional model for a cross-section of the anther wall under plane strain, assuming dehiscence to be driven by epidermal dehydration, which causes changes in the preferred curvature of the wall. We adapt the bilayer model of Nelson *et al.* (2011) to describe these changes in preferred curvature as a function of the turgor pressure of the epidermal cell layer. Endothecial secondary thickening provides a resistance to bending; we assume the bending resistance of the epidermis to be negligible in comparison. Dehydration of the epidermis generates an in-plane tension F^{+*}, transmitting a moment to the endothecium which induces bending. Throughout the forthcoming discussion, stars distinguish dimensional quantities from their dimensionless counterparts. Terms with '$+$' superscripts refer to the epidermis, while those with '$-$' superscripts refer to the endothecium.

S1.1 Governing equations

Assuming that each pair of locules is symmetrical, we restrict attention to one locule. Motivated by experiments, we assume that a small portion at the base of the locule remains undeformed upon dehiscence, providing a fixed support for the upper portion of the locule. The angle of inclination of the anther wall to the locule base is denoted by $\theta(s^*)$, where s^* is a measure of arclength relative to the fixed support (Figure S1). Denoting the curvature of the wall by κ^*, and introducing Cartesian coordinates x^* and y^* oriented as in the figure, we have the following geometric relations:

$$\kappa^* = \frac{\mathrm{d}\,\theta}{\mathrm{d}\,s^*}, \qquad \frac{\mathrm{d}\,x^*}{\mathrm{d}\,s^*} = \cos\theta, \qquad \frac{\mathrm{d}\,y^*}{\mathrm{d}\,s^*} = \sin\theta. \tag{1}$$

Thus, knowing $\kappa^*\,(s^*)$, the locule shape can be reconstructed by integrating (1).

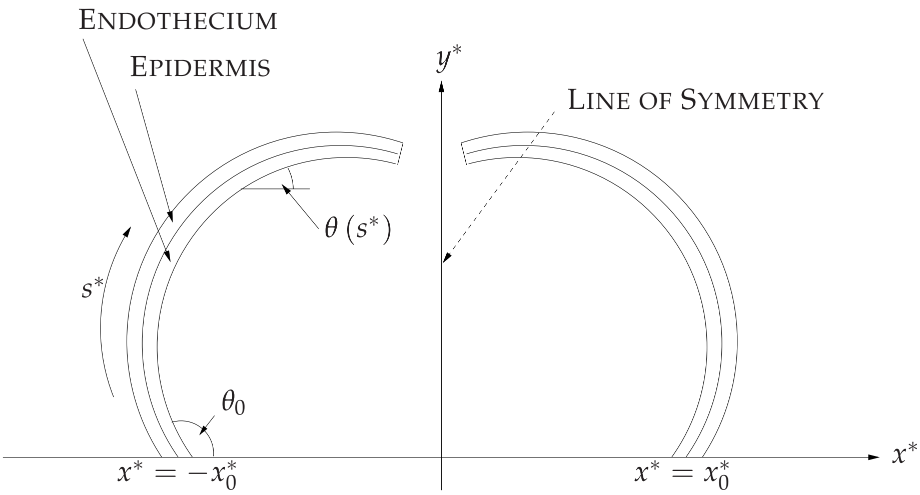

Figure S1: Geometry of the anther segment under consideration, and associated coordinate systems. The composite layer comprises epidermal and endothecial cell layers, which we assume to always remain strongly adhered to one another.

We denote tangential stress resultants in each layer by $F^{\pm *}$, and normal stress resultants in the endothecium by N^*; N^* generates a bending moment M^*. Between the endothecium and the epidermis act a frictional stress Q^* and normal reaction R^*, as shown in Figure S2. Under these assumptions, a balance of forces gives (Nelson *et al.*, 2011)

$$\frac{\mathrm{d}F^{-*}}{\mathrm{d}s^*} + N^*\kappa^* - Q^* = 0, \qquad \frac{\mathrm{d}N^*}{\mathrm{d}s^*} - F^{-*}\kappa^* + R^* = 0. \tag{2}$$

A balance of moments upon the same element, assuming that the contribution of F^{-*} is negligible, gives

$$\frac{\mathrm{d}M^*}{\mathrm{d}s^*} - N^* + Q^*h^* = 0, \tag{3}$$

to leading order, where $2h^*$ is the endothecial thickness. The Q^*h^* term was omitted from the model of Nelson *et al.*; we estimate its magnitude below.

Taking a discrete representation of the upper cell layer, and assuming θ varies slowly between adjacent cells, tangential and normal force balances yield (Nelson *et al.*, 2011)

$$Q^* = -\frac{\mathrm{d}F^{+*}}{\mathrm{d}s^*}, \qquad R^* = -\kappa^* F^{+*}. \tag{4}$$

Introducing the composite in-plane tension $F^* = F^{-*} + F^{+*}$, (2–4) give force and moment equations for the composite layer:

$$\frac{\mathrm{d}F^*}{\mathrm{d}s^*} = -\kappa^* N^*, \qquad \frac{\mathrm{d}N^*}{\mathrm{d}s^*} = \kappa^* F^*, \qquad \frac{\mathrm{d}M^*}{\mathrm{d}s^*} = N^* + h^* \frac{\mathrm{d}F^{+*}}{\mathrm{d}s^*}. \tag{5}$$

We prescribe the following constitutive assumptions, governing the extensions of the cell layers,

$$F^{\pm *} = k^{\pm *}\left(\lambda^{\pm} - \lambda_0^{\pm}\right), \tag{6}$$

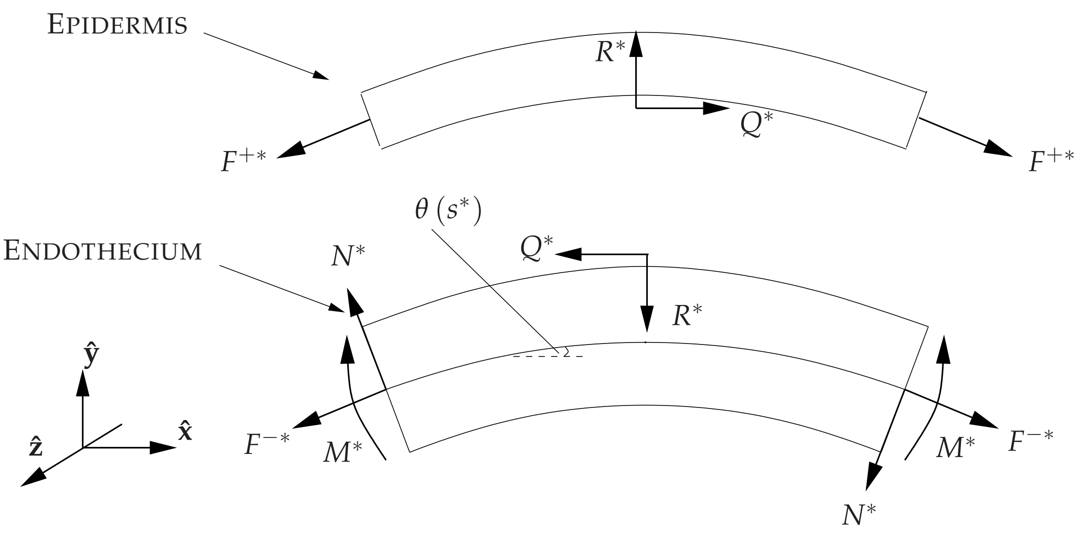

Figure S2: Distribution of forces along an element of the substrate, the resultant stresses and bending moments. Clockwise arrows indicate bending moments acting the negative $\hat{\mathbf{z}}$-direction.

in which $\lambda^{\pm}$ are the in-plane stretches of the two layers, $\lambda_0^{\pm}$ represent the resting strains of the layers, and $k^{\pm *}$ are extensional stiffness parameters. In the endothecium, we consider these quantities to be evaluated on the centreline. Endothecial secondary thickening is incorporated into the model via an increase in k^{-*}. Assuming that the frictional force, Q^*, is sufficient for perfect adhesion between the epidermis and the endothecium, and taking $\kappa^* h^* \ll 1$, we may set $\lambda \equiv \lambda^+ = \lambda^-$ so that the net in-plane tension is given by

$$F^* = \left(k^{+*} + k^{-*}\right)\lambda - \left(k^{+*}\lambda_0^+ + k^{-*}\lambda_0^-\right). \tag{7}$$

In (7), the first term represents the composite extensibility while the second term may be influenced by the hydration of both cell layers through $\lambda_0^{\pm}$.

We make a further constitutive assumption for the endothecium, assuming its bending moment is related to its curvature according to

$$M^* = D^*\left(\kappa^* - \tilde{\kappa}_0^*\right), \tag{8}$$

where D^* is the endothecial resistance to bending (a quantity which we estimate below) and $\tilde{\kappa}_0^*$ represents the preferred curvature of the endothecium in the absence of the epidermis; we assume $\tilde{\kappa}_0^*$ is spatially uniform.

Substitution of (6) and (8) into (5c) gives

$$\frac{\mathrm{d}\,\kappa^*}{\mathrm{d}\,s^*} = \frac{N^*}{D^*} + \frac{\mathrm{d}\,\kappa_0^*}{\mathrm{d}\,s^*}, \tag{9}$$

where $\kappa_0^* = \tilde{\kappa}_0^* + h^* k^{+*}\left(\lambda - \lambda_0^+\right)/D^*$ can be interpreted as an effective preferred curvature of the composite layer, incorporating contraction or expansion of the epidermis. We represent epidermal dehydration by a reduction in λ_0^+, causing κ_0^* to increase.

It is convenient to rewrite the governing equations in terms of the Lagrangian arc-length variable $\tilde{s}^*$, related to s^* via

$$\frac{\mathrm{d}\, s^*}{\mathrm{d}\, \tilde{s}^*} = \lambda. \tag{10}$$

We nondimensionalise the problem by scaling lengths against the resting length of the composite layer (L_0^*), curvatures against $1/L_0^*$ and stress resultants against k^{-*}. Writing $N = N^*/k^{-*}$, equations (5a,b), (9) and (1) then become

$$\frac{\mathrm{d}\, F}{\mathrm{d}\, \tilde{s}} = -\lambda\kappa N, \qquad \frac{\mathrm{d}\, N}{\mathrm{d}\, \tilde{s}} = \lambda\kappa F, \qquad \frac{\mathrm{d}\, \kappa}{\mathrm{d}\, \tilde{s}} = \alpha\lambda N + \Phi\frac{\mathrm{d}}{\mathrm{d}\, \tilde{s}}\left(\lambda - \lambda_0^+\right), \tag{11a}$$

$$\frac{\mathrm{d}\, \theta}{\mathrm{d}\, \tilde{s}} = \lambda\kappa, \qquad \frac{\mathrm{d}\, x}{\mathrm{d}\, \tilde{s}} = \lambda\cos\theta, \qquad \frac{\mathrm{d}\, y}{\mathrm{d}\, \tilde{s}} = \lambda\sin\theta, \tag{11b}$$

with F given by

$$F = (1+\beta)\,\lambda - \left(\lambda_0^- + \beta\lambda_0^+\right). \tag{12}$$

The above system depends upon three dimensionless parameters:

$$\alpha = k^{-*}L_0^{*2}/D^*, \qquad \beta = k^{+*}/k^{-*}, \qquad \Phi = \alpha\beta h^*/L_0^* \equiv k^{+*}L_0^* h^*/D^*, \tag{13}$$

which respectively capture the endothecial resistance to extension relative to its resistance to bending, the extensional stiffness of the epidermis relative to that of the endothecium, and the extent to which dehydration of the epidermis generates changes in the preferred curvature of the anther, resisted by the bending resistance of the endothecium.

S1.2 Boundary conditions

We solve (11–12) subject to boundary conditions appropriate to the three cases described in the main text (see Figure S3). In all cases, we assume a rigid support at $\tilde{s} = 0$ as follows:

$$\theta = \theta_0, \qquad x = -x_0, \qquad y = 0 \qquad \text{on } \tilde{s} = 0, \tag{14}$$

where $x_0 = x_0^*/L_0^*$ is the locule width scaled against the natural length of the anther segment.

In case I, the anther wall is tighly curled with opposite walls in point contact; we apply boundary conditions on the contact point at the symmetry line ($\tilde{s} = \tilde{s}_c$) as follows:

$$\text{Case I:} \quad x = 0, \quad \theta = -\frac{\pi}{2}, \quad F = 0, \qquad \kappa = \tilde{\kappa}_0 + \Phi\left(\lambda - \lambda_0^+\right) \qquad \text{on } \tilde{s} = \tilde{s}_c. \tag{15}$$

The third and fourth boundary conditions in (15) respectively enforce that the vertical force and the bending moment vanish at the contact point. In the region $\tilde{s}_c < \tilde{s} < 1$, the anther wall has constant curvature. Epidermal dehydration reduces λ_0^+, causing the layer to gradually uncurl. When $\tilde{s}_c = 1$ there is a transition to case II, in which the anther remains closed, but it is no longer necessary to track the moving boundary at $\tilde{s} = \tilde{s}_c$. Instead, we impose:

$$\text{Case II:} \quad x = 0, \quad F\sin\theta - N\cos\theta = 0, \qquad \kappa = \tilde{\kappa}_0 + \Phi\left(\lambda - \lambda_0^+\right) \qquad \text{on } \tilde{s} = 1. \tag{16}$$

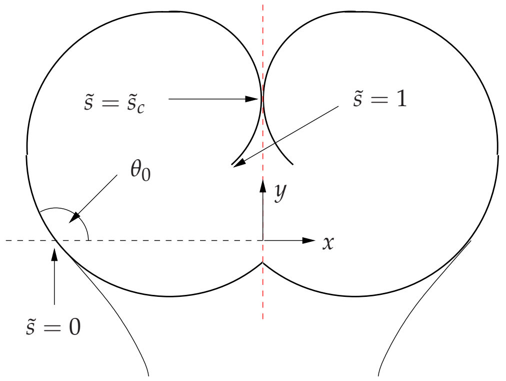

(a) Case I: Anther closed; wall contact is at $\tilde{s} = \tilde{s}_c$

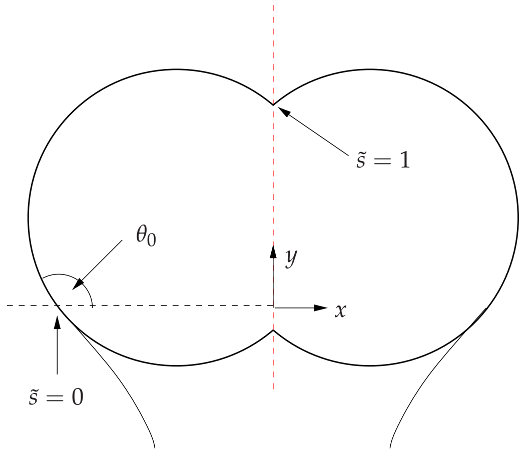

(b) Case II: Anther closed; wall contact is at $\tilde{s} = 1$

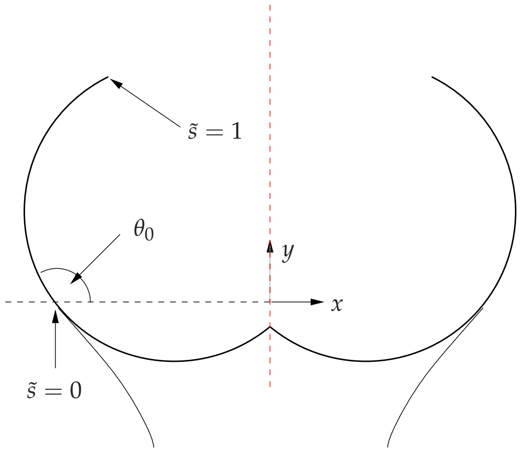

(c) Case III: Anther opens

Figure S3: The three cases considered in the model. Red dotted lines illustrate the line of symmetry between the locules. Black dotted lines demark the upper portion of the locule.

As λ_0^+ decreases further, and the preferred curvature falls, we monitor the horizontal force exerted upon the symmetry boundary, given by

$$F_{contact} = 2F\cos\theta + 2N\sin\theta. \tag{17}$$

Once this force decays to zero, case III applies, in which the boundary at $\tilde{s} = 1$ moves away from the symmetry line, mimicking the opening of the anther. Continued epidermal dehydation (reduction of λ_0^+) generates increasingly open configurations. In case III, all forces and the bending moment vanish at the boundary:

$$\text{Case III:} \qquad F = 0, \qquad N = 0, \qquad \kappa = \tilde{\kappa}_0 + \Phi\left(\lambda - \lambda_0^+\right) \qquad \text{on } \tilde{s} = 1. \tag{18}$$

In this case, the conditions (18) provide a uniformly valid solution of (11a).

S1.3 Parameter estimation

Table 1 summarises the dimensional parameters in the model, and associated physical values for lily and *Arabidopsis* anthers where these are available. We estimate D^* experimentally, as described in Section Notes S2: below; the remaining parameters are estimated via cell-scale models of each layer.

S1.3.1 Cell-scale model of the epidermis

We estimate the epidermal parameters, k^{+*} and λ_0^+, in terms of cell-scale quantities by balancing forces in an epidermal cell. Treating each epidermal cell as a rectangular box of height $2h^{+*}$, with elastic walls extending due to turgor pressure p^{+*}, the in-plane stress resultant is given by

$$F^{+*} = 2E^{+*}\left(\lambda^+ - 1\right) - 2p^{+*}h^{+*}, \tag{19}$$

where E^{+*} is the extensional stiffness of each cell wall. Comparing (19) with (6), we have $k^{+*} = 2E^{+*}$ and

$$\lambda_0^+ = 1 + \frac{2p^{+*}h^{+*}}{k^{+*}} \equiv 1 + P. \tag{20}$$

We regard p^{+*} as the difference between the hydrostatic pressure and the osmotic pressure; as the epidermis dehydrates, it is therefore possible that P may become negative. We, consider solutions starting in a reference state with $\lambda_0^+ > 1$ and allow λ_0^+ to gradually decrease as the epidermis dehydrates.

S1.3.2 Cell-scale model of the endothecium

In contrast to the epidermis, endothecial cell walls have secondary thickening, comprising stiff lignin fibres which form a helix around the cell. These fibres cause the endothecium to resist stretching and bending. We assume that the lignin fibres provide a dominant mechanical contribution to the endothecial cells, although their interaction with the cell wall will undoubtedly be significant. However, we use the secondary thickening alone to estimate the relative resistances of the endothecium to bending and stretching. Cell-scale parameters are summarised in Table S1.

We denote the Young's modulus of the lignin fibres by E_f^*, their Poisson ratio by ν_f, and their radius by R_f^*. Costello (1977) gives the bending stiffness of an isolated helix as

$$D_{cell}^* = \frac{E_f^* \pi R_f^{*4} \sin\gamma}{2(2 + \nu_f \cos^2\gamma)}, \tag{21}$$

where γ is the pitch angle. Endothecial cells are approximately $10 - 20\,\mu$m long with approximately 5 turns of the helix per cell; we calculate the pitch angle to be in the region of $\gamma = 7 - 14°$. Denoting the extensional stiffness of the helix by k_{cell}^*, and using the result of Love (1944), we have

$$k_{cell}^* = \frac{E_f^* \pi R_f^{*4} \sin\gamma}{4h^{*2}}\left(\frac{\cos^2\gamma}{1+\nu_f} + \sin^2\gamma\right). \tag{22}$$

Parameter	Description	Estimated Value
p^{+*}	Turgor pressure of epidermal cells	2 bar
h^{+*}	Half-thickness of epidermal cells	
E^{+*}	Stiffness of epidermal cell walls	
R_f^*	Radius of lignin fibres	25 nm
E_f^*	Young's modulus of lignin fibres	
ν_f	Poisson ratio of lignin fibres	≈ 0.5
γ	Pitch angle of lignin helix	$7 - 14°$

Table S1: Parameters appearing in the cell-scale models. The above epidermal turgor estimate is taken from Bonner & Dickinson (1990). Properties of lignin fibres are based upon measurements of lily anthers.

The quantities D^*_{cell} and k^*_{cell} represent the bending stiffness and extensional stiffness of a single endothecial cell. We expect these values to contribute to the expressions for similar macroscopic quantities for sheets of endothecial cells. From (21) and (22), we estimate α as follows:

$$\alpha = \frac{k^{-*}L_0^{*2}}{D^*} \sim \left(\frac{k^*_{cell}}{D^*_{cell}}\right) L_0^{*2} \sim \frac{L_0^{*2}}{h^{*2}} \gg 1. \tag{23}$$

Equation (23) suggests that endothecial secondary thickening results in a high resistance to stretching and a comparatively low resistance to bending, allowing shrinkage of the epidermis to bend the composite structure. This description of the endothecial cell wall incorporates only the lignin helix and not other cell wall components, nor pre-stress in the cell wall due to endothecial turgor. While there is considerable scope to improve this approximation, we believe the ratio of resistances to bending and stretching predicted above has a sufficient level of accuracy for our purposes.

S1.3.3 The composite layer

Due to the presence of endothecial secondary thickening, we expect the epidermal resistance to extension to be much less than that of the endothecium ($\beta \ll 1$) and the endothecial resistance to extension to be much greater than its resistance to bending ($\alpha \gg 1$). The formula for the dimensionless parameter Φ can be rearranged (using (23)) to give

$$\Phi = \beta\frac{k^{-*}L_0^*h^*}{D^*} \sim \beta\frac{L_0^*}{h^*}, \tag{24}$$

Since $\beta \ll 1$ and $h^* \ll L_0^*$, we conclude that it is appropriate to assume $\Phi = O(1)$, as this captures the essential balance between epidermal contraction and endothecial bending (see (13)). Numerical solutions of the full model (11,12) subject to boundary conditions (14–18) are shown in Figures 2 and 3.

S1.4 Reduced model in the inextensible limit

For $\alpha \gg 1$ and $\beta \ll 1$, such that $\alpha\beta = \Phi/(h^*/L^*) \gg 1$, we rescale dependent variables according to

$$N = \hat{N}/\alpha, \qquad F = \hat{F}/\alpha, \tag{25}$$

reducing (11a) to

$$\frac{\mathrm{d}\hat{F}}{\mathrm{d}\tilde{s}} = -\lambda\kappa\hat{N}, \qquad \frac{\mathrm{d}\hat{N}}{\mathrm{d}\tilde{s}} = \lambda\kappa\hat{F}, \qquad \frac{\mathrm{d}\kappa}{\mathrm{d}\tilde{s}} = \lambda\hat{N} + \Phi\frac{\mathrm{d}}{\mathrm{d}\tilde{s}}\left(\lambda - \lambda_0^+\right). \tag{26}$$

To leading order in α^{-1}, (12) implies that

$$\lambda = \lambda_0^- + \beta\left(\lambda_0^+ - \lambda_0^-\right). \tag{27}$$

Restricting attention to the case in which $\lambda_0^- = 1$ and λ_0^+ is spatially uniform, (26) reduces to the classical beam equations

$$\frac{\mathrm{d}\hat{F}}{\mathrm{d}\tilde{s}} = -\kappa\hat{N}, \qquad \frac{\mathrm{d}\hat{N}}{\mathrm{d}\tilde{s}} = \kappa\hat{F}, \qquad \frac{\mathrm{d}\kappa}{\mathrm{d}\tilde{s}} = \hat{N}, \tag{28}$$

to leading order in β. Boundary conditions follow directly from (15–18), with the leading order condition on κ now being

$$\kappa = \tilde{\kappa}_0 + \Phi\left(1 - \lambda_0^+\right). \tag{29}$$

The system (28,29) is governed by a single material parameter (stretching in (27) decouples from the problem); leading-order configurations are determined by Φ alone, with transitions between cases I, II and III occuring at

$$\lambda_0^+ = 1 + \frac{C_i}{\Phi}, \tag{30}$$

for $O(1)$ constants C_i. In this limit, the shapes of solution branches vary trivially with Φ, as illustrated in Figure 3. Figure S4 illustrates the distributions of stress and strain attained by the configurations shown in Figure 2. The figure confirms that stresses are of order $1/\alpha$ (see (25)) and variations in stretch are of order β (see (27)), with strain varying from approximately 15% extension to approximately 10% compression for the configurations shown (corresponding to a choice of $\beta = 0.2$).

Notes S2: Estimating the forces of anther dehiscence

To determine the forces associated with anther dehiscence, lily anthers were placed upon a flat support and compressed by a prescribed load. The anthers were allowed to dehydrate, and it was recorded whether the force of anther opening was sufficient to overcome the load, or whether the load was sufficient to keep the anther closed.

Mature anthers just prior to opening were removed from the flowers of pink oriental lilies just before testing. Each flower contained six anthers, of which two were used as non-loaded controls. The anthers were placed in the orientation of Figure 1c, on a glass slide (stabilised using blu-tack if necessary) and the load was applied upon the upper surface (equivalent to the x-direction in the model

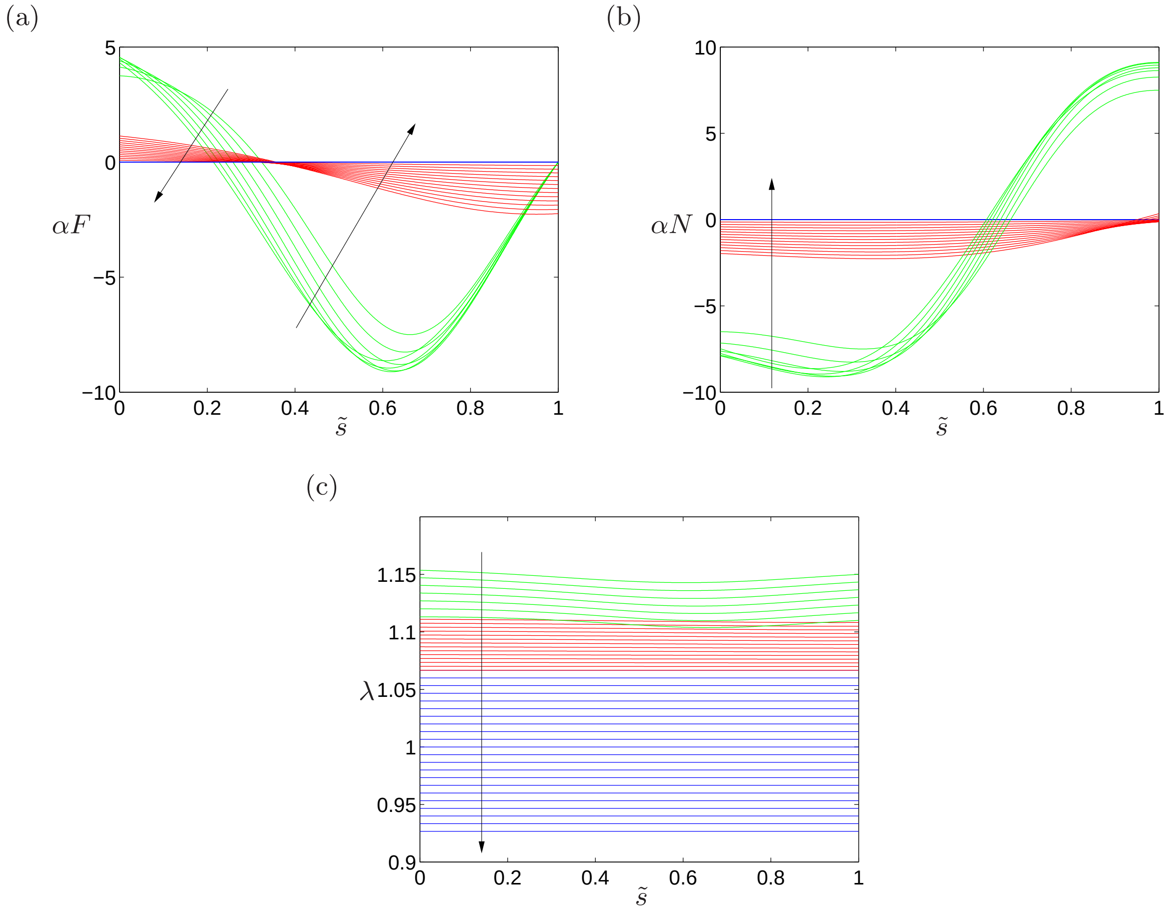

Figure S4: Stress and strain distributions versus arc length, corresponding to the configurations given in Figure 2(a): (a) tangential stress resultant ($F < 0$ represents compression); (b) normal stress resultant; (c) tangential stretch. Compressive stresses in case I (green curves in (a)) are relieved by a snap through to case II (red curves); epidermal dehydration reduces stresses F and N to zero (blue curves, case III).

above) by means of another flat plate upon which varying masses were placed. Masses were distributed along the length of the anther. For each set of six anthers, if both controls had opened (*i.e.* a visible split along the whole length of the anther could be seen) but none of the loaded anthers had opened, then this load was considered great enough to overcome the opening force. A total of 78 anthers were tested and opening times varied from 50 minutes to 6 hours. Mass of the order of 2 g was needed to prevent any loaded anthers opening. Since the mass applied was spread over two locule pairs we can assume that 1 g, *i.e.* a distributed load on each locule pair of approximately 0.01 N, was sufficient force to keep the anther closed.

Section S1.4 suggests that dimensional stress resultants are of magnitude k^{-*}/α, *i.e.* D^*/L_0^{*2}. These stress resultants have dimensions of force per unit length. For a critical force of 0.01 N, distributed over a length of 20 mm (the length of the glass slide), the experimental results suggest stress resultants of approximately 0.5 Pa m. For $L_0^* = 2$ mm (see Table 1), this gives an estimate for D^* of 2×10^{-6} Pa m^3.

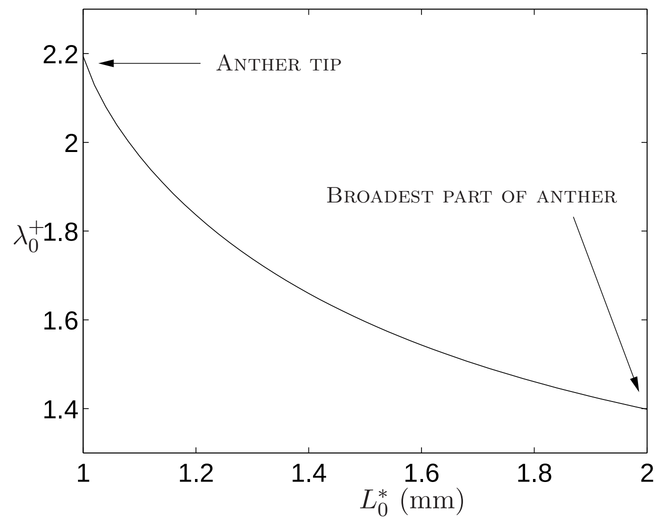

Figure S5: Critical value of λ_0^+ (equivalently epidermal hydration) required for a transition to an open (case III) configuration, as a function of L_0^*. Parameters correspond to lily anthers, as given in Figure 2. At the anther tip, where L_0^* is smallest, less epidermal dehyradtion is required to open the anther.

Comparing (20) and (30) suggests that Φ is of magnitude $k^{+*}/(2p^{+*}h^{+*})$ when the anther opens. Noting (13), and assuming that the two cell layers are of approximately equal thickness ($h^{+*} \simeq h^*$), it follows that

$$D^* \sim 2p^{+*}L_0^*h^{*2}. \tag{31}$$

For the values of h^* and L_0^* estimated in Table 1, and an estimated epidermal turgor pressure of approximately 2 bar (Bonner & Dickinson, 1990), the theoretical model gives $D^* \sim 2 \times 10^{-6}$ Pa m^3, which is consistent with the experimental prediction, providing a consistency check for our model assumptions.

Notes S3: The effects of variations in L_0^*

As Figure 1a illustrates, during lily anther dehiscence, the tips of the anther open before the remainder of the anther. Taking measurements of the lily anthers of Figure 1 (in both closed and open configurations) suggests that the width (x_0^*) of the closed anther remains approximately uniform along the anther's length, while the natural length of the locule wall (L_0^*) falls to approximately half of its maximal value near the tips. These changes in L_0^* affect the parameters x_0, α and Φ in the model, leading to a change in the degree of dehydration required to open the anther. Figure S5 shows the critical level of epidermal hydration (λ_0^+) required to open the anther, as a function of L_0^*, for parameters corresponding to a lily anther as in Figure 2. As L_0^* is varied, α and Φ are updated according to (13), keeping material parameters fixed. The figure illustrates that for smaller values of L_0^*, the anther will open for larger values of λ_0^+, *i.e.* requiring less epidermal dehydration. The model thus illustrates that dehiscence initially occurs at the tips as a consequence of the shape of the anther wall.

References

Bonner L, Dickinson H. 1990. Anther dehiscence in *Lycopersicon esculentum* II. *New Phytologist*, **115**: 367–375.

Costello GA. 1977. Large deflections of helical spring due to bending. *ASCE J. Eng. Mech. Div.*, **103** (3):418–487.

Love AEH. 1944. *A treatise on the mathematical theory of elasticity*. Dover publications Inc. New York, USA.

Nelson MR, Howard D, Jensen OE, King JR, Rose FRAJ, Waters SL. 2011. Growth-induced buckling of an epithelial layer. *Biomechanics and Modeling in Mechanobiology*, **10**:883–900.
